# Micro simulated moving bed chromatography-mass spectrometry as a continuous on-line process analytical tool

**DOI:** 10.1007/s00216-023-05023-9

**Published:** 2023-11-10

**Authors:** Juliane Diehm, Lennart Witting, Frank Kirschhöfer, Gerald Brenner-Weiß, Matthias Franzreb

**Affiliations:** Institute of Functional Interfaces, Karlsruhe Institute of Technology, Hermann-von-Helmholtz-Platz 1, 76344 Eggenstein-Leopoldshafen, Germany

**Keywords:** µSMB-MS, Process analytical technology (PAT), Continuous buffer exchange, Sample pre-processing, Desalting, Native ESI-MS

## Abstract

Continuous manufacturing is becoming increasingly important in the (bio-)pharmaceutical industry, as more product can be produced in less time and at lower costs. In this context, there is a need for powerful continuous analytical tools. Many established off-line analytical methods, such as mass spectrometry (MS), are hardly considered for process analytical technology (PAT) applications in biopharmaceutical processes, as they are limited to at-line analysis due to the required sample preparation and the associated complexity, although they would provide a suitable technique for the assessment of a wide range of quality attributes. In this study, we investigated the applicability of a recently developed micro simulated moving bed chromatography system (µSMB) for continuous on-line sample preparation for MS. As a test case, we demonstrate the continuous on-line MS measurement of a protein solution (myoglobin) containing Tris buffer, which interferes with ESI-MS measurements, by continuously exchanging this buffer with a volatile ammonium acetate buffer suitable for MS measurements. The integration of the µSMB significantly increases MS sensitivity by removing over 98% of the buffer substances. Thus, this study demonstrates the feasibility of on-line µSMB-MS, providing a versatile PAT tool by combining the detection power of MS for various product attributes with all the advantages of continuous on-line analytics.

**Graphical abstract:**

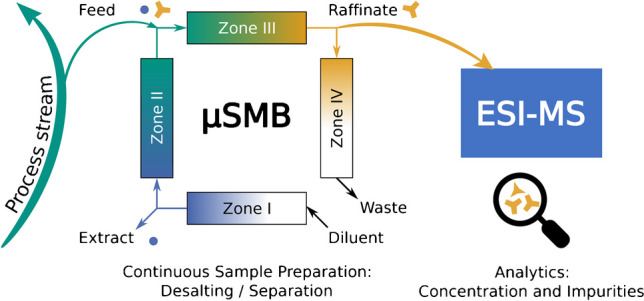

**Supplementary Information:**

The online version contains supplementary material available at 10.1007/s00216-023-05023-9.

## Introduction

Continuous manufacturing is state of the art for many chemical processes [1], offering numerous advantages over batch processing, such as faster time-to-market, lower cost of goods, a more consistent product quality without batch-to-batch variability, or a lower carbon footprint and is therefore of interest to the (bio-)pharmaceutical industry [2, 3]. However, active process control is crucial for successful continuous processing due to potential variations in process parameters and input materials [3]. The limited number of process observations combined with long analysis times for biomolecules has been identified as a critical challenge for the next generation of biomanufacturing [4].

As a result, there has been extensive research into process analytical technology (PAT) in recent years [3]. In the field of biopharmaceuticals, spectroscopic methods, such as UV/Vis spectroscopy, near-infrared spectroscopy, or Raman spectroscopy, have received considerable attention as they can perform in-line measurements without the need for sample preparation while also featuring a fast measurement time [5, 6]. In contrast, commonly used off-line analytical methods, like high-performance liquid chromatography (HPLC) or mass spectrometry (MS), are less considered because they either have a longer processing time (HPLC) or require sample preparation (MS) [6].

Nevertheless, the application of MS as a PAT tool is promising [7], as it is considered one of the most powerful techniques [8] capable of detecting a wide range of quality attributes, including glycosylation patterns [9, 10], charge variants [11, 12], aggregates [13, 14], as well as process-related impurities such as host cell proteins [15–17].

Wang et al. demonstrated the potential of direct analysis in real time (DART)-MS as a PAT tool, not only for process analytics [18] but also for accelerating process development [19]. However, the DART method is suitable primarily for small and medium-sized molecules [20, 21]. For biological molecules, so-called native electrospray ionization (ESI)-MS measurements are of particular interest, as the structural information is preserved during the measurement [13]. It is mainly applied in combination with liquid chromatography for sample pre-processing (LC-MS), since most biological buffer systems are incompatible with ESI-MS, as they are either non-volatile or introduce significant noise [22, 23]. Miniaturized LC systems are particularly promising, having a shorter analysis time [8]. This is crucial, as it increases the time available to make process adjustments, if necessary, to keep all critical quality attributes (CQAs) within the specified range [3]. Therefore, CQAs should ideally be measured continuously. In the case of LC-MS, the MS measures samples at a high acquisition rate and LC is the time-limiting step. Although existing systems are often referred to as online LC-MS (due to the direct coupling of the two systems), they still rely on batch-wise LC pre-processing, which hinders true on-line implementation in terms of PAT. To address this limitation, the batch-wise LC sample pre-processing needs to be replaced by a continuous (chromatographic) method. Although continuous implementations of chromatography processes exist at a preparative scale, they have not been applied for analytical purposes so far, as the existing equipment is not designed to handle small volumes and is therefore not suitable for analytical purposes.

To overcome this limitation, we recently miniaturized simulated moving bed (SMB) chromatography, a widely used continuous chromatography technique, to the micro-scale (µSMB) [24]. This study demonstrates its effectiveness as an analytical tool for continuous on-line sample preparation coupled with on-line native ESI-MS measurements. In typical SMB processes, a series of identical chromatography columns is divided into four zones of different mobile phase flow rates by adding and withdrawing material flows between the columns. Additionally, the chromatography columns are switched in the opposite direction of the mobile phase flow such that a time discrete countercurrent movement between stationary and mobile phase results. If the flow rates in each zone as well as the column switching time interval are selected accordingly, a continuously added feed stream can be separated into two fractions, the raffinate and the extract stream [25]. Here, each zone fulfills a specific task: zone 1 is for the regeneration of the stationary phase, zones 2 and 3 are for the separation of the components in the feed stream, and zone 4 is for the regeneration of the mobile phase [26]. For further details on the SMB principle, we refer to the literature [25–27].

In this proof-of-concept study, we performed a continuous buffer exchange of a protein solution based on size-exclusion chromatography (SEC) with the µSMB system. We then hyphenated the µSMB to an ESI-MS for continuous protein detection. We selected Tris buffer as an example of a biological buffer that interferes with MS measurements and continuously replaced it with a volatile ammonium acetate buffer. Myoglobin (Mb) was chosen as a protein because its non-covalently bound prosthetic heme group allows easy verification of its structural integrity during measurements [28, 29].

## Materials and methods

### Chemicals and buffers

All chemicals were purchased from Sigma-Aldrich (St. Louis, USA). Myoglobin from equine skeletal muscle had a purity of 95–100%, ammonium acetate of >99.95%, and Tris-HCl of > 99%. Buffers were prepared with ultrapure water (Milli-Q Gradient, Merck Millipore, Burlington, USA) and degassed in an ultrasonic bath for at least 30 min before usage. Stock solutions of 100 mM NH_4_OAc (pH 7), 1 mM Tris (pH 7), and 10 g/L Mb in 10 mM NH_4_OAc (pH 7) were prepared. Feed solutions for the µSMB experiments were prepared from these stock solutions.

### Mass spectrometry

Mass spectrometry was performed on an X-500R QTOF mass spectrometer (AB Sciex LLC, Framingham, USA). Instrument handling and data acquisition were performed using SCIEX OS Software (version 2.2, AB Sciex LLC, Framingham, USA). Data processing, such as extraction of extracted-ion chromatograms, calculation of mean spectra, and baseline chromatogram extraction, was performed using the built-in Explorer Tool. A peak width of *m*/*z* ± 0.02 was used for all molecules to extract extracted-ion chromatograms. The baseline chromatogram was extracted with an *m*/*z* ratio between 400 and 500. Protein reconstructions from spectra were performed using the Bio Tool Kit of the Explorer Tool with a limited input *m*/*z* range from 1700 to 2600. The output mass range was chosen from 5 to 20 kDa with a step mass of 0.5 Da.

#### Operating conditions

All MS measurements were performed in TOF-MS mode with positive polarity. The TOF mass range was between *m*/*z* 400 and *m*/*z* 2600 with an accumulation time of 0.25 s. The spray voltage was set to 5000 V and the declustering potential to 40 V. The collision energy was 20 V for native measurements and 40 V for non-native measurements. Ion source gas 1 (nitrogen) was set to 50 psi, curtain gas to 25, and CAD gas to 7. The ion source temperature was set to 100 °C. The mass spectrometer was equilibrated for at least 45 min and calibrated with an external standard (ESI positive solution for the Sciex X500B system, Sciex) before a measurement was started.

#### Off-line measurements

Samples containing 10 µg/mL Mb in 10 mM NH_4_OAc buffer with varying Tris concentrations of 0, 1, 5, 10, and 100 mM were measured off-line in duplicate. A syringe pump (Harvard Apparatus Model 11 (55-1111), Holliston, USA) was used to continuously infuse the sample solutions into the ESI source. The flow rate was set to 30 µL/min, corresponding to the raffinate flow rate of the µSMB experiments. Each sample was measured for 2 min; only the second minute was used for data evaluation to ensure that the system was flushed with the respective sample. The sample without Tris was measured under native and non-native conditions. All other samples were measured under native conditions only.

### µSMB process

**Fig. 1:**
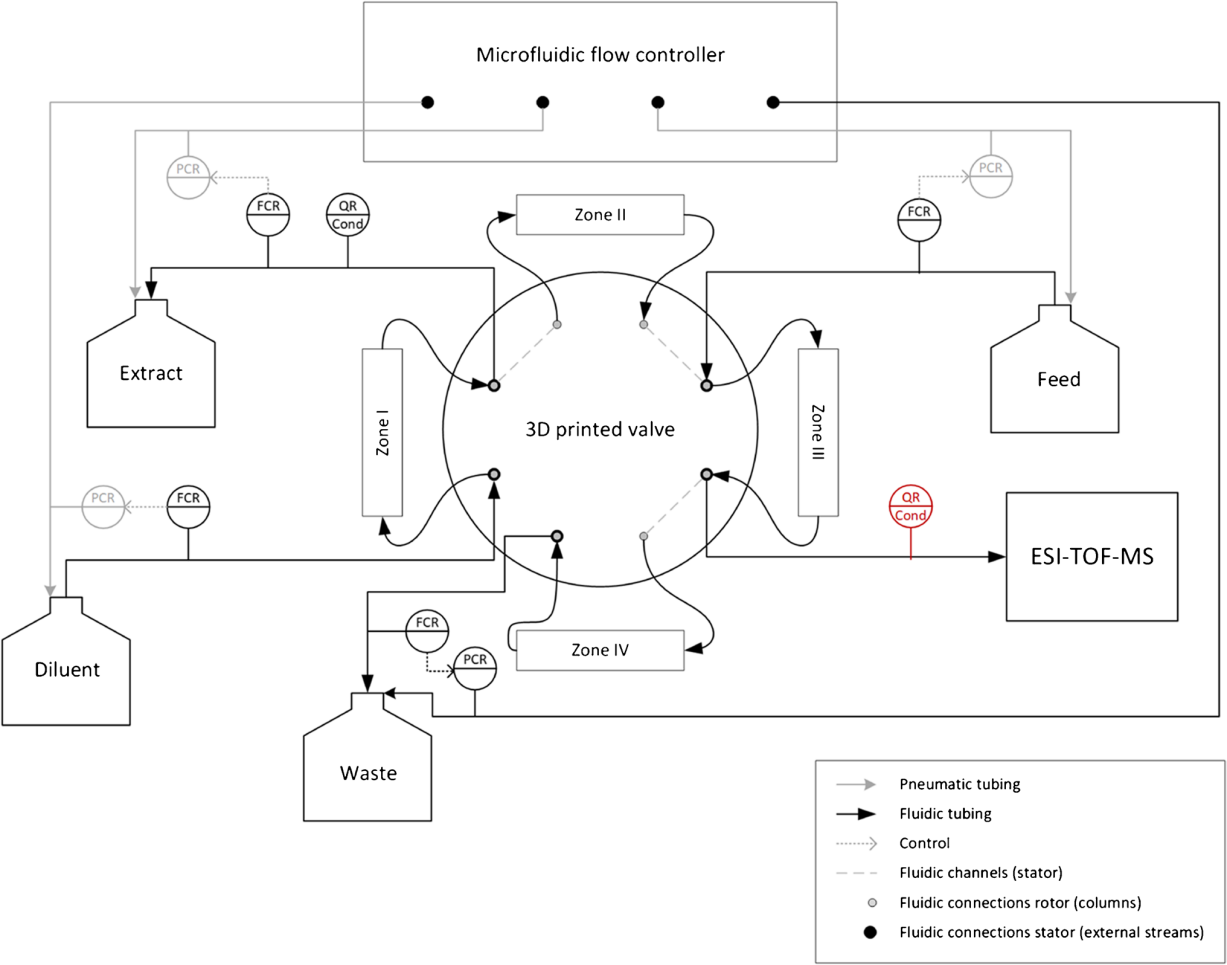
Instrumentation and piping diagram of the used µSMB setup. The conductivity sensor in the raffinate stream is only used when the µSMB is not coupled to the MS

The µSMB system used is a four-zone open-loop setup with one column per zone (Fig. 1). This means that the buffer leaving the fourth zone is not recycled into the first zone. Theoretically, the fourth zone of the µSMB system is not required in this case, as it is not necessary to regenerate the mobile phase. We still decided to use four zones because this decouples the flow rate of zone 3 from the raffinate flow rate. Therefore, this setup enables users to freely choose and independently select the flow rate in zone 3 during process point development, as well as the option to set the raffinate flow rate to a specified fixed value (30 µL/min in this case). Alternatively, this could be achieved with a diverting valve, but since this would have decreased the analyte concentration in the raffinate stream, we chose the four-zone open-loop setup. The raffinate flow rate is of particular interest as it is infused into the mass spectrometer. The µSMB system consists of a 3D printed central rotary valve, which is actuated by a stepper motor (pan drive PD60-3-1161, TRINAMIC Motion Control GmbH & Co. KG, Hamburg, Germany). The valve was fabricated according to Diehm et al. [30], with the only difference that a PTFE sheet (PTFE Virginalfolie 0.25 V AD, Hightechflon GmbH & Co. KG, Konstanz, Germany) was used for sealing instead of a silicone sheet. The in- and outlets of the chromatography columns were connected to the valve’s rotor, and the system’s in- and outlet streams were connected to the stator. A pressure-driven microfluidic flow controller (OB1 M3K+, Elveflow, an Elvesys brand, Paris, France) in combination with flow sensors (SLI-1000, Sensirion AG, Stäfa, Switzerland) was used for closed-loop control of the flow rates. Conductivity sensors (ÄKTApurifier, Cytiva Life Sciences, Uppsala, Sweden) with conductivity meters (Labor-Konduktometer 703, Knick Elektronische Messgeräte GmbH & Co. KG, Berlin, Germany) were applied for on-line conductivity monitoring in the extract and raffinate streams. The conductivity sensors were calibrated to determine the Tris concentration with solutions containing 0, 1, 5, 10, 50, and 100 mM Tris. The resulting slopes of the calibration curves were 15.359 mM/(mS/cm) for the extract and 15.382 mM/(mS/cm) for the raffinate, with an intercept of 0.3656 mM and 0.2993 mM, respectively. The conductivity sensor in the raffinate stream was only used for stand-alone µSMB experiments to reduce the delay volume between µSMB and mass spectrometer.

The general setup of the µSMB has been described previously [24]. However, in this study, some modifications had to be made for coupling to the MS system: As the raffinate is directly infused into the ESI source, it is not possible to directly control the outlet pressure of this stream and therefore it is not possible to control the raffinate’s flow rate with the microfluidic flow controller. The flow rates of all other streams, including the waste stream, were controlled instead so that the raffinate stream’s flow rate is adjusted automatically following the system’s overall liquid flow mass balance. An instrumentation and piping diagram of the setup with all fluidic and pneumatic connections is shown in Fig. 1.

A Matlab application with a graphical user interface was developed with Matlab’s AppDesigner (R2022a, MathWorks Inc., Natick, USA) to control the flow rates and valve switching, as well as to record the sensor data of the flow and conductivity sensors and the applied pressures of the flow controller.

#### Chromatography columns

Four Omnifit BenchMark Microbore chromatography columns (3×50 mm, column volume approx. 353 µL, Diba Industries Inc., Danbury, USA) with 0.2-µm stainless steel end pieces were packed with Sephadex G-10 buffer exchange resin (Cytiva Life Sciences, Uppsala, Sweden) for use in the µSMB experiments. The slurry was prepared according to the manufacturer’s specifications. Two columns were connected with a pack adapter and prefilled with the slurry before being connected to an ÄKTA pure 25 M system (Cytiva Life Sciences, Uppsala, Sweden). The columns were compressed by increasing the flow rate from 25 to 200 µL/min with a step size of 25 µL/min. Each step was held for 10 min and purified water was used as the mobile phase. Afterward, the pack adapter was replaced by an end piece and the column was again flushed for at least 40 min at a flow rate of 200 µL/min.

Next, the column and particle porosities were determined with tracer experiments. For this purpose, 2.5 µL of 1% (v/v) acetone (total porosity) or 1 g/L blue dextran (column porosity) were injected at a flow rate of 100 µL/min with water as mobile phase. Experiments were performed in triplicate for all columns. In addition, the retention times of Mb and Tris were determined by injecting 2.5 µL of a solution containing 1 g/L Mb and 10 mM Tris. Furthermore, the experiments were performed with a zero dead volume connector instead of a chromatography column to assess the system’s contribution to the retention times of the different species.

#### Process point determination

A first estimate of a suitable process point was determined with the triangle theory [25], using the retention times of Mb and Tris and the calculated porosities of the single-column experiments. This process point was then used as the starting point for a process point optimization with the software CADET-SMB (version 2.12, CADET version 3.2.1, Institute of Bio- and Geosciences 1 (IBG-1) of Forschungszentrum Jülich (FZJ), Germany) [31, 32]. For this, a single-column model was needed. The lumped rate model with pores was selected, as preliminary simulations showed that it is not possible to distinguish the film transfer coefficient from particle diffusion based on the single-column experiments. Thus, the general rate model has no advantage over the lumped rate model with pores, but has significantly longer computational times. The axial dispersion coefficient *D*_ax_ was estimated based on the correlation of Chung and Wen (1) [33], the film transfer coefficient *k*_film_ by the correlation of Wilson and Geankoplis (2) [34], and the molar diffusion coefficient *D*_m_ according to Polson (3) [35] with the particle Reynolds number *Re*_p_ (4) [36], the mean particle diameter of the chromatography medium *d*_p_, the interstitial velocity *u*_int_, the density of water *ρ*_H2O_, the dynamic viscosity of water *η*_H2O_, the interstitial porosity of the chromatography column *ε*_int_, and the molecular weight *M*_w_ of species *i*. The differential evolution algorithm of CADET-SMB was used for the process point optimization. An overview of all input parameters is given in Table S1 in the Supplementary Information (SI).

1 $${D}_{\mathrm{ax}}= \frac{{{u}_{\mathrm{int}}\cdot d}_{\mathrm{p}}\cdot {\epsilon }_{\mathrm{int}}}{0.2+0.011\cdot {\left({\epsilon }_{\mathrm{int}}\cdot R{e}_{\mathrm{p}}\right)}^{0.48}}$$

2 $${k}_{\mathrm{film},i}=\frac{1.09\cdot {D}_{\mathrm{m},i}}{2\cdot {r}_{\mathrm{p}}\cdot {\varepsilon }_{\mathrm{int}}}\cdot {\left({\epsilon }_{\mathrm{int}}\frac{{u}_{\mathrm{int}}\cdot {d}_{\mathrm{p}}}{{D}_{\mathrm{m}, i}}\right)}^{0.33}$$

3 $${D}_{\mathrm{m},i}=2.74\cdot {10}^{-9}\cdot {{M}_{\mathrm{w},i}}^{-\frac{1}{3}}$$

4 $$R{e}_{\mathrm{p}}=\frac{{u}_{\mathrm{int}}\cdot {d}_{\mathrm{p}} \cdot {\rho }_{{\mathrm{H}}_{2}\mathrm{O}}}{{\eta }_{{\mathrm{H}}_{2}\mathrm{O}}}$$

#### µSMB experiments

All µSMB experiments were performed with the parameters determined with the process point optimization (diluent flow rate: 122 µL/min, extract flow rate: 44 µL/min, feed flow rate: 15 µL/min, raffinate flow rate: 30 µL/min, waste flow rate: 63 µL/min, switching time: 120 s). The microfluidic flow controller was operated with a feed-forward closed-loop control. Thus, initial values for the set pressures of the different process streams are required. These were determined by replacing the chromatography columns with capillaries with equivalent back pressure and starting the flow control with the desired flow rates as flow setpoints and 0 mbar for all pressure setpoints. The determined pressure start values were 1300 mbar for the diluent, 750 mbar for the extract, 1000 mbar for the feed, and 600 mbar for the waste. The µSMB system with the chromatography columns was equilibrated with 10 mM NH_4_OAc buffer for at least 45 min before each experiment. Then, the feed reservoir was filled with the respective feed solution and the experiment was started. After the experiment was finished, the system was first flushed with water and then with 20% (v/v) ethanol for storage.

A first µSMB experiment was performed stand-alone, without coupling to the mass spectrometer, with a feed solution containing 100 mM Tris.

### µSMB-MS

To hyphenate µSMB and MS, the raffinate outlet port was directly coupled to the ESI interface of the MS system. To keep the delay volume between µSMB and MS as low as possible, the conductivity sensor of the raffinate was not used in this setup. Both, the µSMB and mass spectrometer, were equilibrated similarly to the stand-alone experiments. Two different experiments were performed: In the first one (run 1), the concentration of Tris in the feed solution was increased stepwise, while the concentration of Mb was kept constant at 0.2 g/L and the concentration of NH_4_OAc was 10 mM. Ten millimolar NH_4_OAc was also used as diluent. Tested Tris concentrations were 0, 1, 5, 10, 50, and 100 mM. The first section without Tris in the feed was run for eleven cycles (44 column switches) to ensure that the system runs stable. For all other concentrations, three cycles were conducted. The MS measurement continued during the exchange of the feed solution to the next higher Tris concentration, while the µSMB experiment had to be stopped.

The second experiment (run 2) was performed with a constant Tris concentration of 10 mM and Mb concentration of 0.2 g/L in the feed solution and was run for six cycles. All other conditions were identical to the first run.

## Results and discussion

Current applications of off-line MS measurements in biopharmaceutical processing range from the detection of aggregates, charge variants, and other product-related impurities to the detection of process-related impurities. In our proof-of-concept study, native Mb (holoMb) represents the product, and dissociated Mb (apoMb) alongside the heme group exemplifies product-related impurities. We first verified that we are able to detect holoMb, apoMb, and heme with the MS measurement and evaluated the influence of Tris buffer, as one example of a frequently used biological buffer, on the measured signals. Subsequently, we tested whether we could reduce the negative influence of the Tris buffer on the MS measurements by hyphenating the µSMB to the mass spectrometer.

### Off-line MS measurements

#### Myoglobin

**Fig. 2:**
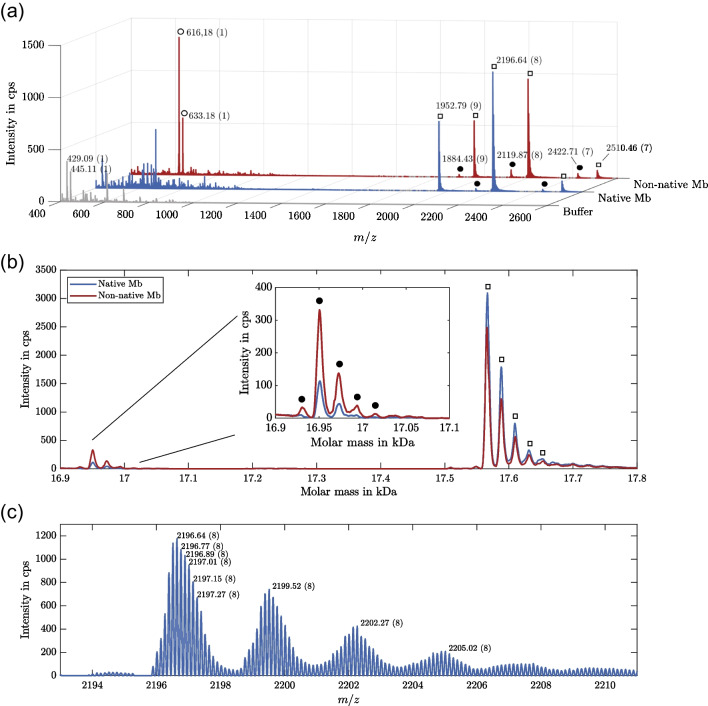
Measurement of Mb in native and non-native state. (**a**) background spectrum of 10 mM NH_4_OAc buffer and spectra of Mb under native and non-native measurement conditions. (**b**) Protein reconstruction of the Mb spectra from (**a**). (**c**) Close up of the Mb main peak with isotopic peaks and sodium adducts. In (**a**) and (**c**), charge states are assigned in parenthesis after *m/z* labels. In (**a**) and (**b**), peaks of holoMb are marked with white squares, of apoMb with black circles, and of heme with white circles

First, we performed off-line measurements with pure Mb samples to ensure that we can detect all target molecules under native conditions. The resulting spectra are depicted in Fig. 2(a). The first row shows a background spectrum of the used NH_4_OAc buffer, the second row shows a spectrum of Mb under native conditions, and the last row shows the same Mb sample under non-native measurement conditions. Comparing the buffer and the native Mb spectrum, five additional peaks with three different charge states, ranging from sevenfold to ninefold, are detected. These signals are probably caused by holoMb and apoMb. Comparing these peaks with the non-native spectrum, it can be observed that the signal intensity of the peaks with the lower *m*/*z* ratio of each charge state increases while the signal intensity of the peaks with the higher *m*/*z* ratios decreases. It can therefore be concluded that the peaks with higher *m*/*z* ratios are holoMb (marked with white squares in the spectra) and the peaks with lower *m*/*z* ratios are apoMb (marked with black circles in the spectra).

This assumption was verified by reconstructing the protein masses from the spectra; the resulting spectrum is depicted in Fig. 2(b). Two peak groups are detected; the main peak of the first one has a molar mass of 16950.5 Da and the second one of 17566.0 Da. This is in accordance with the molar masses of apoMb and holoMb reported in the literature [28, 37, 38], confirming their identity and our ability to measure both variants of Mb. The difference between the measured peaks is 615.5 Da, which corresponds to the mass of the dissociated heme group.

The signal of the heme group can also be observed in the non-native Mb spectrum in Fig. 2(a) at *m*/*z* 616.18 and 633.16 (oxidized form) [39]. Remarkably, the heme signal has a higher intensity than the apoMb signal, although it is formed by the dissociation of holoMb to apoMb and therefore must be present at the same concentration as apoMb. However, the mass of the heme group is more than ten times smaller than that of apoMb, and since smaller molecules are generally easier to ionize, the higher signal intensity is due to a higher ion yield.

Figure 2(b) shows that there are not two single peaks detected for apoMb and holoMb, but rather two peak groups. The difference in molar mass of the individual peaks is approximately 23 Da, which equals the mass of sodium. Therefore, the peaks in one group correspond to different sodium adducts of the respective molecule.

The close-up of the main holoMb peak in Fig. 2(c) shows that these sodium adducts were already present in the original native Mb spectrum in Fig. 2(a). Furthermore, each sodium adduct peak consists of multiple isotopic peaks. Therefore, in order to use all detected signals of holoMb and apoMb in the analysis of the following experiments, the integral of the protein reconstruction spectrum for the respective peak group was usually used instead of an extracted-ion chromatogram of a single signal.

#### Influence of Tris concentration

Most biological buffers are incompatible with ESI, as they are either not volatile or introduce a high level of noise to the background signal [22, 23]. Therefore, the influence of different concentration levels of Tris buffer on the MS detection of Mb was investigated next with off-line measurements. For this purpose, the concentration of Tris in a Mb solution was varied between 0 and 1 mM, as these levels of Tris buffer have been previously introduced into ESI-MS systems [22].

**Fig. 3:**
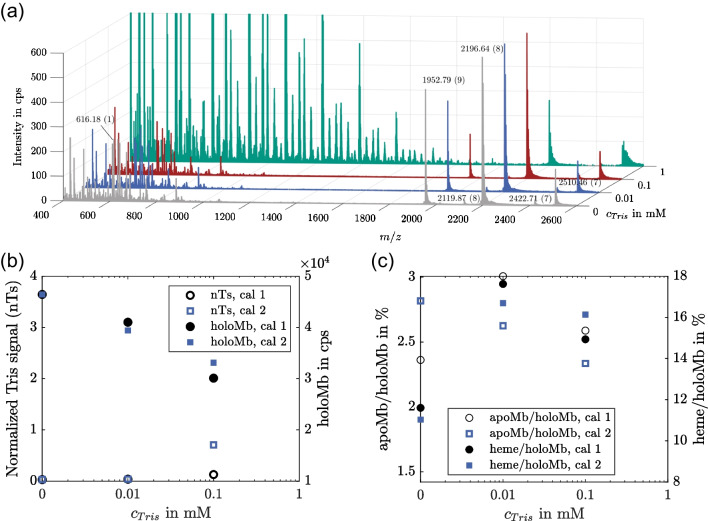
Influence of Tris buffer on MS detection of Mb. (**a**) Mass spectra of solutions containing 10 µg/mL Mb with increasing Tris concentrations. Charge states are assigned in parenthesis after *m/z* labels. (**b**) Normalized Tris signal (*m/z* 593.23) and amount of detected holoMb in dependence of Tris concentration. (**c**) Influence of Tris concentration on detected percentage of apoMb and heme group. The detected amount of apo/holoMb was calculated by integration of the respective peaks in the protein reconstruction chromatogram

The resulting spectra in Fig. 3(a) show no visible effect at a Tris concentration of 0.01 mM for the signals of holoMb with seven- and eightfold charge states, while the peak height of the holoMb signal with ninefold charge state slightly decreases. Starting at a concentration of 0.1 mM, the peak heights of all apoMb signals decrease. In addition, a shift towards higher sodium adducts is observed, especially for the peak corresponding to the sevenfold charged species. This effect is further enhanced in the sample containing 1 mM Tris, suggesting that sodium is a contaminant in the Tris buffer. Also, the detected amount of Mb decreases further and even the most intensive holoMb signal at *m*/*z* 2196.64 has a decreased peak height, indicating that the sensitivity of the mass spectrometer is negatively affected. Furthermore, as previously observed, Tris induces a lot of noise in a wide range of *m*/*z* ratios, with the most intensive signal at *m*/*z* 593.23 [22]. This signal reaches up to 23 kcps (a full-scale plot is included in Fig. S1 in the SI) which is 50 times higher than the detected signal level of the heme group, thus hindering its detection.

Figure 3(b) depicts the calibration curve of the measured Tris signal at *m*/*z* 593.23 ± 0.2. The calibration was measured twice with independent calibration solutions and the Tris signal was normalized with the baseline chromatogram of the respective measurement. The Tris signal shows an exponential increase with concentration in the logarithmic plot, corresponding to limited growth. This is typical for an analytical system when its detection limit is reached and not only indicates that the applied concentrations are much too high, but also prevents the calculation of the exact Tris concentration from the signal. Therefore, at these concentrations, it is no longer possible to reconstruct the Mb concentration by taking the Tris concentration into account, which emphasizes the strong influence of Tris concentrations as low as 1 mM (speaking from a bioprocessing point of view, where concentrations up to 10 mM and higher are state of the art) on the measurement quality.

Figure 3(b) also depicts the detected signal level of holoMb. As mentioned above, the amount of holoMb was calculated by integrating the respective peaks of the protein reconstruction chromatogram. This way, all charge variants and sodium adducts are included in the calculation and effects such as the shift to higher sodium adducts with higher Tris concentrations are not considered. Nevertheless, the total detected amount of holoMb decreases exponentially with the Tris concentration. Starting from 46.4 kcps at 0 mM Tris, the detected amount is more than 2.5 times lower at a Tris concentration of 1 mM, meaning less than 40% is detected. Even at a Tris concentration of 0.01 mM, the detection level is below 90%, underlining the severe impact on the measurement quality even at comparatively low buffer concentrations.

Figure 3(c) shows the influence of the Tris concentration on the detection of apoMb and the heme group, respectively, as two different ways to detect the product-related impurity level of our model system. While the amount of apoMb is derived from the protein reconstruction, the amount of heme was calculated with the extracted-ion chromatogram at *m*/*z* 616.16. Overall, the amount of heme is higher than that of apoMb, which is again caused by the better ion yield. In both cases, the detected level increases at low Tris concentrations before decreasing at higher Tris concentrations. This can either mean that low Tris concentrations induce the dissociation of holoMb or increase the ion yield of the molecules. Excluding the point without Tris, both detection routes show comparable results with a drop in apoMb and heme level detection of approx. 10% from 0.01 to 0.1 mM Tris and of approx. 56% from 0.1 to 1 mM Tris.

These results clearly show that the applied ESI-MS measurements are not a suitable analytical method for samples containing Tris buffer without prior treatment. Even at concentrations as low as 0.01 mM, an effect of Tris on the measured overall concentrations of holoMb, apoMb, and heme is observable. Other parameters, like the signal intensity of the Mb main Peak at *m*/*z* 2196.64, are less influenced by Tris and only show a decreased signal intensity starting at a Tris concentration of 1 mM, which is still far below the concentrations typically applied in biotechnological processes.

### Desalting with the µSMB

In the following µSMB-MS experiments, we increased the Tris concentration to higher levels up to 100 mM, which are more relevant for bioprocessing. Therefore, it is important to ensure that the µSMB provides a sufficient desalting level to prevent damage to the MS instrument during the hyphenation experiments. Thus, the desalting performance of the µSMB system was assessed stand-alone before the actual µSMB-MS experiments were performed.

**Fig. 4:**
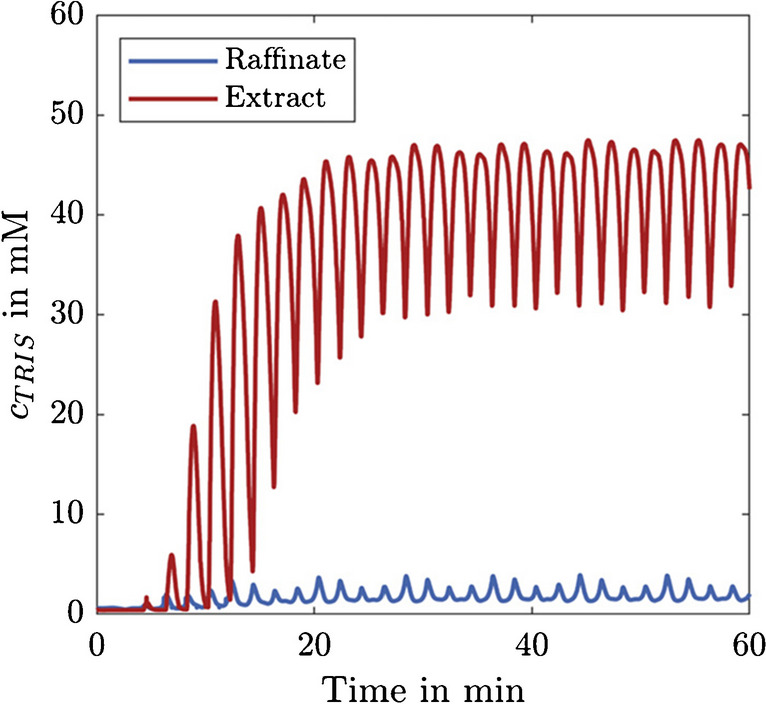
Tris concentrations in the raffinate and extract streams during a µSMB experiment with 100 mM Tris in the feed solution

Figure 4 depicts the measured Tris concentration in the raffinate and extract streams during an experiment with a feed solution containing 100 mM Tris. Tris is detectable in both the extract and raffinate streams; however, the mean concentration in the raffinate is more than 20 times lower compared to the extract, proving that the desalting process is working.

The concentration profiles show the typical startup behavior of SMB processes; after approx. 20 min, a cyclically recurring peak pattern is observed. This state is referred to as cyclic steady state (CSS) and is caused by column switching. Even when the CSS is reached, not all Tris peaks have the same height, but every fourth peak is comparable. This is caused by slight performance differences between the four chromatography columns.

Since the CSS is of particular interest for continuous long-term applications, the mean desalting level was calculated for the CSS rather than for the entire process. This way, the calculated desalting level is also independent of the process time. The calculated mean Tris concentration in the raffinate in the CSS was 1.93 mM, which equals a desalting level of 98.07%.

While this already reduces the Tris concentration by almost two orders of magnitude, it is still below the desalting levels of above 99% previously achieved with the µSMB system [24]. However, these experiments were performed with a different separation system, and a comparison of the single-column chromatograms of both separation systems (see Fig. S2 in the SI) shows that the lower desalting level is caused by a lower single-column performance and not by the µSMB setup.

Comparing these results with the influence of Tris on the MS measurements in the previous section shows that the µSMB is able to remove Tris to a point where it does not damage the MS system. Nevertheless, for the µSMB-MS experiments, we expect to see an influence of Tris on the MS measurements when working with feed concentrations of 100 mM Tris, which is further investigated in the following sections.

### µSMB-MS

#### Hyphenation

The first step for hyphenating the µSMB with the MS system is to generate the desired flow rates for the in- and outlet streams of the µSMB. For the µSMB-MS, it is not possible to directly control the raffinate flow rate with the pressure-driven microfluidic flow controller, as it is not possible to directly control the outlet pressure. Instead, the waste flow rate was controlled to achieve the desired raffinate flow rate based on the overall mass balance of the system.

**Fig. 5:**
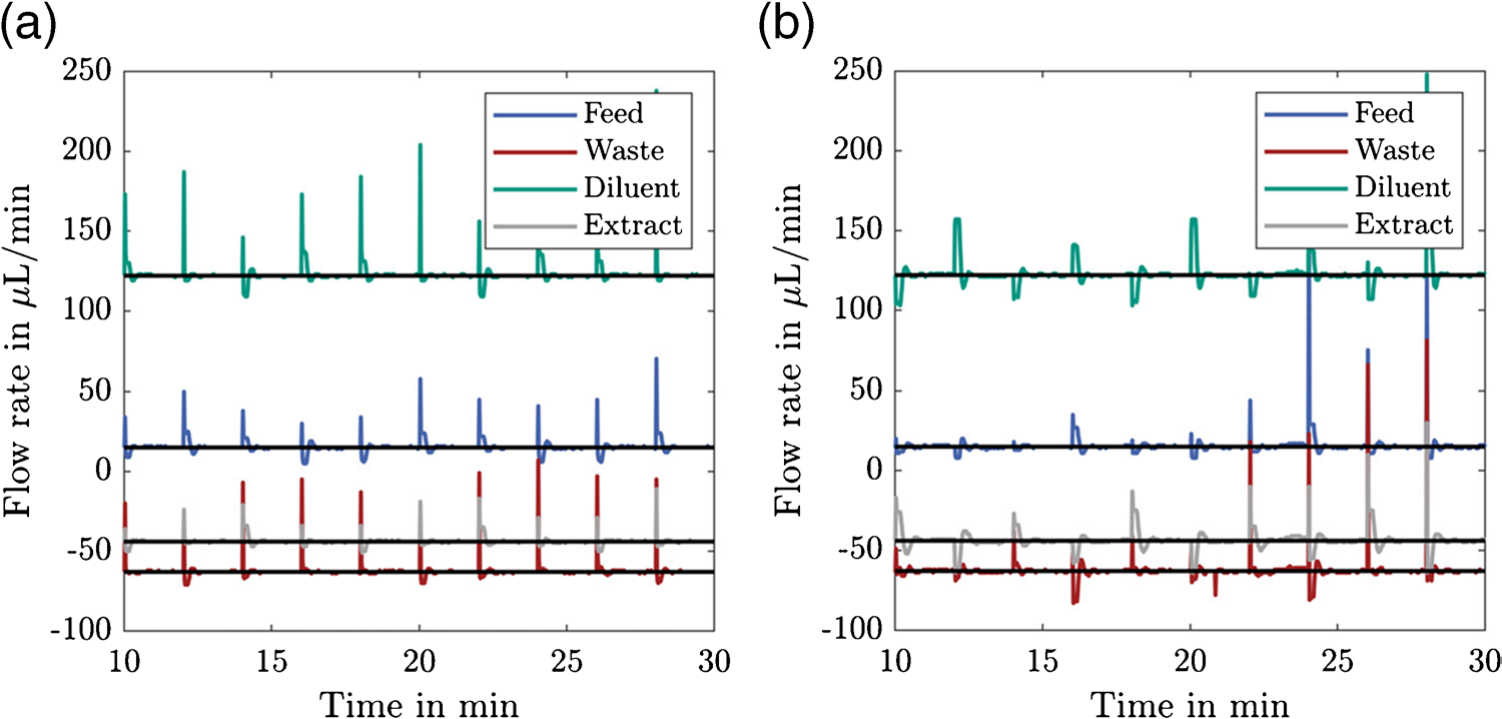
Comparison of the different in- and outlet flow rates without (**a**) and with (**b**) direct coupling of the raffinate stream to the ESI interface of the MS system

This method for flow rate control was first validated without hyphenation to the MS system, the resulting flow rates are given in Fig. 5(a). The horizontal black lines are the respective set flow rates. Positive flow rates correspond to streams entering the system and negative ones are leaving the system. Every 2 min, during the switching process, there is a spike in the flow rates. This has been reported previously and usually has no negative effect on the separation performance due to the short time frame of occurrence [40]. In between the switching processes, the set flow rates are reached, proving the validity of the approach used.

In Fig. 5(b), the raffinate stream is directly infused into the ESI interface of the MS system. This setup is particularly challenging for the flow rate generation, as the raffinate outlet pressure is subject to additional pressure fluctuations caused by the vacuum pumps of the MS system. Some flow rate spikes have a different shape, others are higher compared to the setup without MS, and especially in the waste stream there are some minor flow rate fluctuations between the switches. Nevertheless, the mean flow rate over one switching interval corresponds to the set flow rate, ensuring that each of the SMB zones can fulfill its task. Thus, it is possible to hyphenate the µSMB and MS system hardware-wise.

#### Proof-of-concept

Two different buffer exchange experiments were performed with the µSMB-MS setup to assess whether the hyphenation can improve the MS measurement quality. In the first one (run 1), the Tris concentration in the feed solution was increased step-wise, such that the impact of Tris on the MS equipment could be assessed before further increasing the concentration. The second experiment (run 2) was performed with a constant Tris concentration in the feed.

**Fig. 6:**
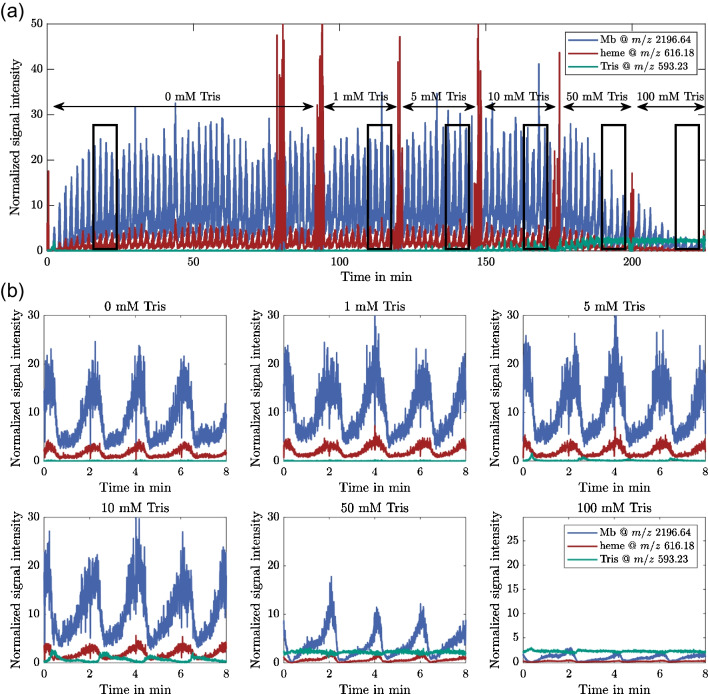
Normalized signals of Mb, heme group, and Tris (**a**) during the entire first µSMB-MS experiment (run 1); (**b**) for the third process cycle at every Tris concentration (marked with black boxes in (**a**))

Figure 6(a) shows the normalized extracted-ion chromatograms of holoMb (*m*/*z* 2196.64 ± 0.02), the heme group (*m*/*z* 616.18 ± 0.02), and Tris (*m/z* 593.23 ± 0.02) throughout the experiment (run 1). The sections with different Tris concentrations are indicated in the plot. During the experiment, the flow had to be stopped several times to increase the Tris concentration in the feed solution. This resulted in an abrupt drop of the baseline signal, which caused a steep increase of the heme signal, as there are still traces of the analytes at the ESI interface and the heme group has a better ionization efficiency than the other molecules. During the phase with 0 mM Tris in the feed solution, the flow had to be stopped one additional time due to a pressure leakage in the feed reservoir. The third SMB cycle of every Tris concentration is depicted in Fig. 6(b) for better comparability. The respective areas are marked with black rectangles in Fig. 6(a). Again, the typical startup behavior of SMB processes is observed. The CSS is already reached after 15 min, since only the raffinate signal is displayed, which reaches its CSS earlier than the extract signal. The peak heights of the Mb signal peaks vary throughout the experiment. As discussed previously for the Tris signal (see Fig. 4), this is caused by differences in the chromatography columns. The first Tris signals are detected at a feed concentration of 5 mM. The peak height increases at 10 mM, where it becomes apparent that the breakthrough of Tris is countercyclical to the detection of Mb. This is interesting for on-line analytical applications, as the Tris concentration is the lowest when most Mb is eluted, resulting in less interference from Tris with the measurement compared to a constant Tris concentration with the mean value.

Up to 10 mM Tris, no adverse effects on the detection of Mb and the heme group are observed. This is in accordance with the stand-alone MS experiments depicted in Fig. 3(a), as no negative effect on the detection of the holoMb main signal was observed for Tris concentrations below 1 mM. At a higher Tris concentration of 50 mM, the detected concentrations of Mb and heme decrease significantly, similar to the previous experiments. Interestingly, the Tris signal does not increase much compared to 10 mM Tris due to the baseline correction: With an expected desalting level of 98%, the average Tris concentration in the raffinate of the 50 mM Tris µSMB experiment is 1 mM. This concentration results in a high noise level for smaller *m*/*z* ratios, as depicted in Fig. 3(a). Consequently, the baseline signal, which is recorded at *m*/*z* ratios between 400 and 500, is also affected. At such high Tris concentrations, it is not only increased, but it also fluctuates with the Tris concentration, which is why the Tris signal no longer shows a cyclic pattern at concentrations above 50 mM. This effect increases further for the 100 mM experiment. Here, the baseline signal is almost ten times higher than in the experiment without Tris. The equivalent of Fig. 6(b) without baseline correction is included in Fig. S3 in the SI for reference.

While we see a significant effect of Tris concentrations exceeding 50 mM on the holoMb main peak detection, we do not observe any negative effects up to a concentration of 10 mM. Therefore, we chose this concentration for a second experiment (run 2), which was performed with a constant Tris concentration in the feed solution. When using higher Tris concentrations or when investigating other features like the ratio of holoMb to apoMb, the desalting level should be increased by adapting the µSMB system, e.g., by using more than one column per zone, by increasing the single column resolution, with further process point optimization or by applying other SMB-process variants.

**Fig. 7:**
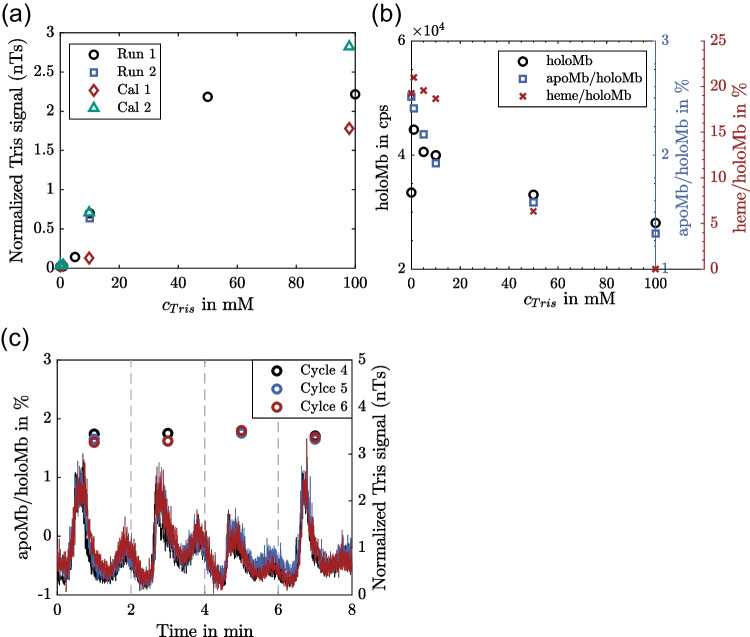
(**a**) Normalized Tris signal as a function of the Tris concentration in the feed of the µSMB experiments. Depicted values are the mean values of the third cycle of each concentration. The Tris concentrations of the calibrations were multiplied with the expected desalting level of 98% in order to match the *x*-axis. (**b**) Detected apoMb percentage and detected amount of Mb as a function of the feed Tris concentration during the experiments. The values are mean values of the third cycle of the µSMB process for each concentration. (**c**) Detected apoMb percentage and Tris signal level for three consecutive cycles (4–6) of µSMB run 2 with a constant Tris concentration of 10 mM in the feed stream. The apoMb level was calculated for each switching interval, as indicated by the dotted vertical lines

Figure 7(a) depicts the mean Tris signal of the third cycle of each Tris concentration of µSMB run 1 for quantitative assessment of the results. In addition, the measured signals from the calibration experiments (Fig. 3(b)) are included for comparison. The concentrations of the calibration standards were multiplied by the expected desalting level to match the *x*-axis. Furthermore, the mean Tris signal of the third cycle of µSMB run 2 is depicted. The results of both µSMB runs are in good agreement, demonstrating that the µSMB-MS system can achieve reproducible desalting levels and that the performance is independent of the buffer concentration during the startup processes. This is important for PAT applications where variations in product quality need to be detected regardless of the previous process state. Although the measurements of the calibration standards are subject to variation, there is good agreement between the experiment and the calibration, meaning that the expected desalting level of 98% was achieved. This shows that the coupling of the MS system to the µSMB has no negative effect on the latter’s performance.

Figure 7(b) shows the effect of the Tris concentration on the MS sensitivity for run 1. Again, for very low Tris concentrations, there is an effect that increases the detected levels of holoMb and heme. At higher concentrations, the detected holoMb concentration and the amount of apoMb and heme decrease exponentially. While the detected apoMb is in the expected range from the calibration results, the detected heme level spans a much broader range, which indicates that the apoMb level is more reliable. This was expected, as Tris induces more noise in the detection *m/z* range of heme. For both the detected amount of holoMb and apoMb, the influence of Tris is less than would have been predicted for a desalting level of 98%, as both signals decrease by only 10% up to a Tris feed concentration of 10 mM. This could be caused by the positive influence of very low Tris concentrations or the effect of the alternating breakthrough of Tris and Mb described in the previous section. Either way, the detection level of both holoMb and apoMb is highly increased by the hyphenation of the µSMB to the MS.

The results in Fig. 7(a) and (b) all refer to just one cycle of the µSMB process for the respective concentration. For a continuous on-line analytical application, the system must provide a reliable and comparable performance over a long time. Therefore, Fig. 7(c) shows the detected ratio of apoMb to holoMb and the normalized Tris signal for three consecutive SMB cycles of run 2. The percentage of apoMb was calculated for one switching time, as indicated by the dotted vertical lines. The curves of the Tris signal again show differences in the separation performance of the different columns. At the same time, the desalting performance of the same column in different cycles is highly reproducible.

Due to the chosen scale of the plot, it can be observed that there are not one but two Tris peaks per switching time, meaning that there are two different concentration fronts of Tris in SMB zone 3 that break through at different times. The fact that the MS system is able to reproducibly capture the dynamic of the Tris breakthrough and the different peak shapes shows that there are no memory effects for the MS measurement that interfere with the long-term measurement. In addition, it is a general indicator of the robustness of the µSMB-MS setup and proves that the µSMB system can provide stable flow rates. The mean detected apoMb percentage remains consistent across switches and cycles, which is reasonable since the mean Tris concentration is also constant. If the apoMb level is calculated over a time period shorter than one switching interval, it is subject to variation (see Fig. S4 in SI), because the Tris concentration is not constant. However, if the same time intervals are used for the calculation in all switches, the obtained results are highly reproducible.

## Conclusion

In this study, we successfully hyphenated a µSMB to an ESI-MS system, demonstrating that the µSMB is a valuable tool for on-line sample pre-processing. Throughout the study, we achieved consistent desalting levels of 98% with the µSMB, improving the MS sensitivity significantly. While the detected level of holoMb already dropped below 40% at a Tris concentration of 1 mM without hyphenation to the µSMB, 89% of holoMb still was detected at a 10 mM Tris concentration when the MS system was coupled to the µSMB. The advantage of the µSMB compared to conventional LC systems is not limited to the continuous mode of operation; using the countercurrent principle additionally leads to a high separation efficiency even with poor performance of the single chromatography columns. This makes the µSMB an interesting tool for many applications and challenging separation problems. In addition to SEC, SMB is frequently applied with various other chromatography modes, e.g., for enantioseparation [25]. Future applications of the µSMB-MS principle are not limited to process control at a preparative scale, but can also provide useful insights during process development. At the same time, the system presented here is not restricted to the SMB principle; the setup can be easily adapted to any type of multi-column chromatography and used to hyphenate it to MS, by simply exchanging the 3D printed valve that is used to connect the columns with one fitting the needs of the particular process.

In order to exploit the full potential of µSMB-MS as a PAT application, future studies should investigate the system’s response time and residence time distribution, a critical aspect for process optimization. The expected response time of the system is between the µSMB switching time and the cycle time, depending on the phase of the SMB cycle in which a fluctuation occurs, i.e., 2–8 min for the system presented in this study, which is comparable to current LC-MS systems [41]. However, the continuous sampling omits the possibility of undersampling and ensures that each fluctuation is accurately captured with respect to its starting point. In addition, further down-scaling of the µSMB system, e.g., by using capillary columns, promises to further reduce the response time and thus increase the applicability of the system.

### Supplementary Information

Below is the link to the electronic supplementary material.

Supplementary file1 (PDF 502 KB)

## Data Availability

The datasets generated and analyzed during the current study are available from the corresponding author on reasonable request.
