# Supplementary material for: Micro simulated moving bed chromatography-mass spectrometry as a continuous on-line process analytical tool
Source: Anal Bioanal Chem. 2023 Nov 10;416(2):373–86. doi: 10.1007/s00216-023-05023-9 (PMC10761468; doi:10.1007/s00216-023-05023-9)
Supplement: Supplementary file 1 — Supplementary file1 (PDF 502 KB) [file 216_2023_5023_MOESM1_ESM.pdf]

Supplementary Information

Micro Simulated Moving Bed Chromatography – Mass Spectrometry as Continuous On-line Process Analytical Tool

Juliane Diehm[1], Lennart Witting[1], Frank Kirschhöfer[1], Gerald Brenner-Weiß[1], Matthias Franzreb[1]

Analytical and Bioanalytical Chemistry

This PDF file includes:

Table S1:	Overview of all model parameters of the SMB process point optimization with CADET-SMB.
Figure S1:	MS spectra of solutions containing 10 µg/mL Mb and different concentrations of Tris.
Figure S2:	Single-column experiments for the separation of Mb and Tris with a Sephadex G10 column and for the separation of bovine serum albumin (BSA) and ammonium sulfate (AS) with a Sephadex G25 fine column.
Figure S3:	MS signals of Mb, heme and Tris during the third SMB process cycle for each Tris concentration of µSMB run 1. The depicted signals are not normalized with the baseline signal.
Figure S4:	Detected apoMb level and normalized Tris signal for five consecutive switching intervals of µSMB run 2 (10 mM Tris in feed solution). The apoMb level was calculated over an interval of 24 s, as indicated by the dotted lines.

[1] Institute of Functional Interfaces, Karlsruhe Institute of Technology, Eggenstein-Leopoldshafen, 76344, Germany; E-Mail: matthias.franzreb@kit.edu

Table S*1* gives an overview of all required input parameters for the process point optimization with CADET-SMB.

Table S1 Overview of all model parameters of the SMB process point optimization with CADET-SMB. SV: start value; LL: lower limit; UL: upper limit.

**Parameter**	**Name in CADET**	**Value**
Column length	opt.column.Length	5.0e-02 m
Column diameter	opt.columnDiameter	3.0e-03 m
Particle radius	opt.particleRadius	5.5e-05 m
Bed porosity	opt.porosityColumn	0.376
Particle porosity	opt.porosityParticle	0.547
Accessible porosity	opt.poreAccessibility	Mb: 0.043<br>Tris: 1
Number of components	opt.nComponents	2
Axial cells per columns	opt.nCellsColumn	40
Radial cells per particle	opt.nCellsParticle	4
Dispersion coefficient	opt.dispersionColumn	1.27e-6 *Q [mL/min]<br>+4,92e-10
Film transfer coefficient	opt.filmDiffusion	Mb: 2.20e-05 m^2/s<br>Tris: 7.78e-05 m^2/s
Binding model	opt.BindingModel	'NoBinding'
Initial particle loading	opt.initialSolid	0 mol/m^3
Discretization in time	opt.timePoints	1000
Number of zones	opt.nZone	4
Number of columns	opt.nColumn	4
Switching time	opt.switch	SV: 103 s; LL: 80 s; UL: 300 s
Q1	-	SV: 2.5e-09 m^3/s;<br>LL: 3.3e-10 m^3/s;<br>UL: 2.5e-09 m^3/s
QF	-	SV: 2.5e-10 m^3/s;<br>LL: 2.5e-10 m^3/s;<br>UL: 2.5e-09 m^3/s
QE	-	SV: 9.7e-10 m^3/s;<br>LL: 3.3e-10 m^3/s;<br>UL: 2.5e-09 m^3/s
QR	-	5e-10 m^3/s
Interstitial velocity	opt.interstitialVelocity	Calculated from volumetric flow rates
Optimization function	$y = 50 \cdot Yield_{Tris,Raf} + (1 - Yield_{Mb,Raf})$	

Fig. S1 shows comparably the MS spectra of Mb solutions with different Tris content. For a 1 mM Tris concentration, the highest Tris signal at 593.23 Da is more than ten times higher compared to the Mb signal at 2196.64 Da.

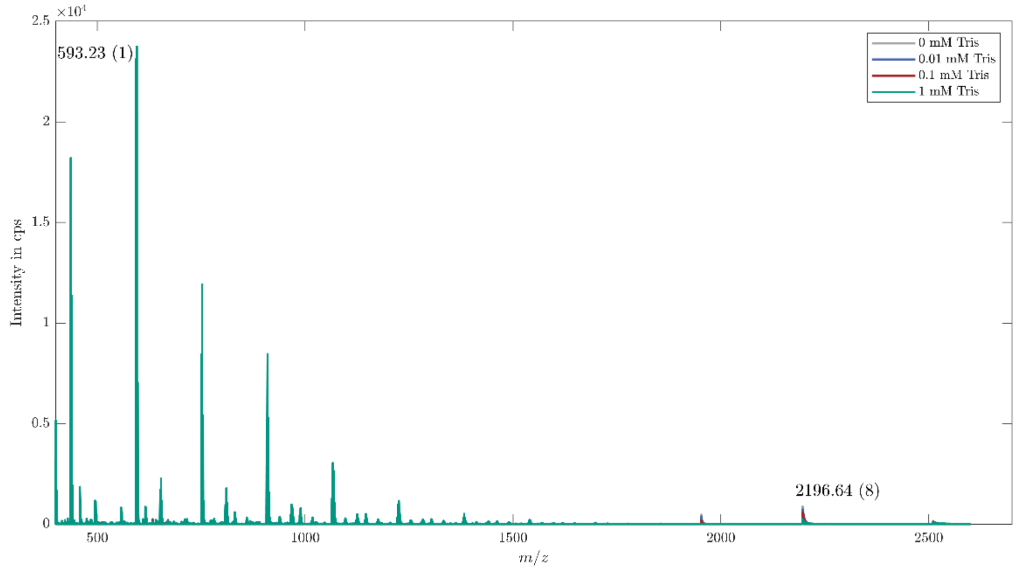

Fig. S1 MS spectra of solutions containing 10 µg/mL Mb and different concentrations of Tris

Fig. S2 shows the single-column desalting experiments of the Mb/Tris system that was used in this study in comparison to the bovine serum albumin (BSA)/ammonium sulfate (AS) system that was applied in a previous study. The peak height of AS was normalized to match that of Tris for better comparability. The retention time difference of the peak maxima is higher for the BSA/AS separation system. In addition, the resolution at the beginning of the peaks is much better. BSA starts to elute before AS, while Mb and Tris almost elute at the same time. The resolution at the beginning of the separation is most important for the raffinate purity. As the desalting performance of the single-column experiment already is much lower for the Mb/Tris system, it is probably not possible to match the µSMB desalting level of the BSA/AS system with further process point optimization. Rather it would be required to change the separation system itself or the µSMB setup instead. Possible approaches are an increased number of columns per zone, longer chromatography columns or the usage of a stationary phase with higher separation performance.

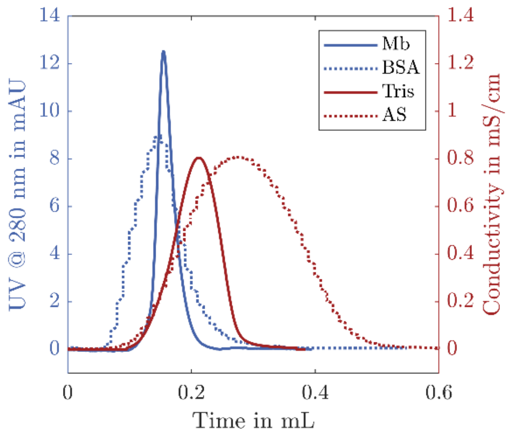

Fig. S2 Single-column experiments for the separation of Mb and Tris with a Sephadex G10 column and for the separation of bovine serum albumin (BSA) and ammonium sulfate (AS) with a Sephadex G25 fine column. For better comparability, the peak height of the AS peak was normalized to match the Tris peak

Fig. S3 depicts the MS signals of Mb, heme and Tris during the third SMB cycle for each Tris concentration of run 1 without baseline correction of the signals. The drop of the Mb signal at Tris concentrations of 50 mM and 100 mM is not as high as with the baseline correction, still the negative influence of Tris on the measurement is clearly observable.

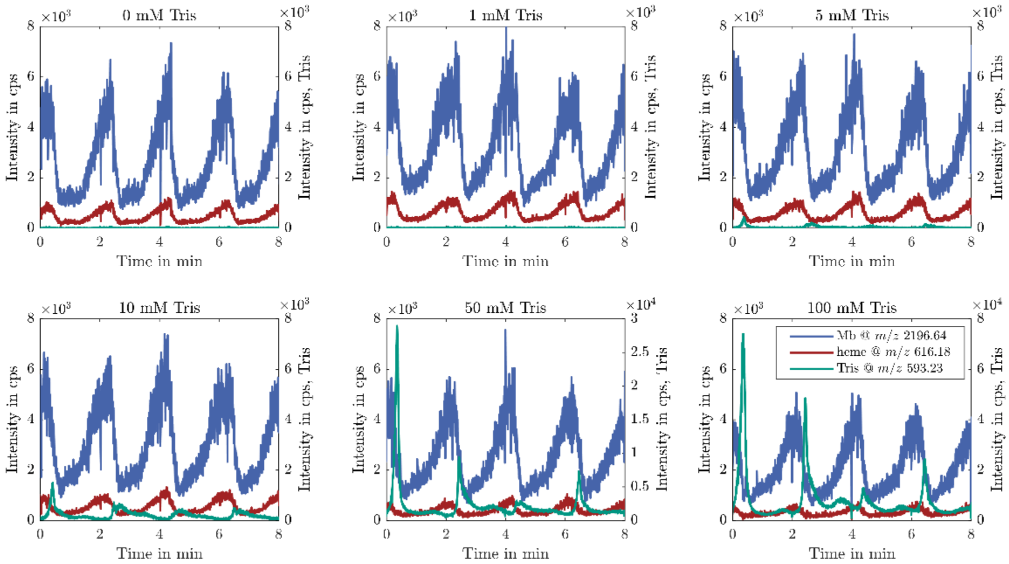

Fig. S3 MS signals of Mb, heme and Tris during the third SMB process cycle for each Tris concentration of µSMB run 1. The depicted signals are not normalized with the baseline signal

Fig. S4 shows the apoMb level and Tris concentration over five consecutive switches in µSMB-MS run 2. The switches were divided into five subsections (24 s per subsection) and the mean detected apoMb percentage was calculated for each subsection. As the interval that was used to calculate the apoMb level is smaller than one switching interval, the influence of the fluctuations in Tris concentration on the detected apoMb level are observable. Despite the differences of the columns, the mean detected apoMb percentage is comparable between the switches for the different subsections. This is favorable for possible on-line analytical applications, as it shows that different time intervals of the SMB process are comparable as long as they are compared to an equivalent time interval of another switch or cycle. A comparison between different subintervals is not possible, as the detected apoMb percentage differs. Interestingly, there is no direct correlation between the Tris concentration and the detected apoMb level, e.g. the detected apoMb level is highest in the third subsection, while the Tris concentration is lowest in the first one. This again suggests that there are is no proportional relationship between the detected apoMb level and the Tris concentration and further investigation is required to clarify the exact effect.

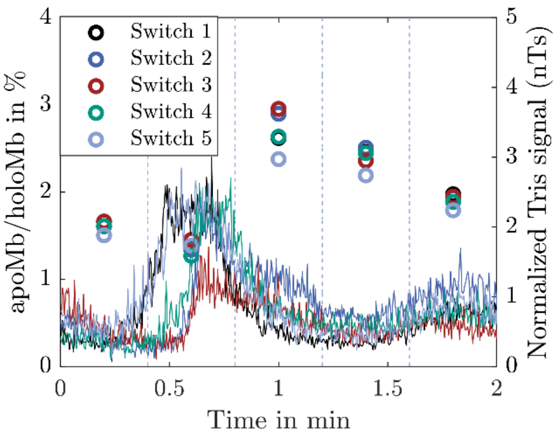

Fig. S4 Detected apoMb level and normalized Tris signal for five consecutive switching intervals of µSMB run 2 (10 mM Tris in feed solution). The apoMb level was calculated over an interval of 24 s, as indicated by the dotted lines
